# Comparing the Drop Vertical Jump Tracking Performance of the Azure Kinect to the Kinect V2

**DOI:** 10.3390/s24123814

**Published:** 2024-06-13

**Authors:** Patrik Abdelnour, Kevin Y. Zhao, Athanasios Babouras, Jason Philip Aaron Hiro Corban, Nicolaos Karatzas, Thomas Fevens, Paul Andre Martineau

**Affiliations:** 1 Faculty of Medicine and Health Sciences, McGill University, 3605 Rue de la Montagne, Montreal, QC H3G 2M1, Canada; patrik.abdelnour@mail.mcgill.ca (P.A.); kevin.zhao2@mail.mcgill.ca (K.Y.Z.); nicolaos.karatzas@mail.mcgill.ca (N.K.); 2 Division of Orthopaedic Surgery, McGill University Health Centre, 1650 Cedar Ave, Montreal, QC H3G 1A4, Canada; jason.corban@mail.mcgill.ca; 3 Department of Experimental Surgery, McGill University, 845 Sherbrooke St W, Montreal, QC H3A 0G4, Canada; athanasios.babouras@mail.mcgill.ca; 4 Department of Computer Science and Software Engineering, Concordia University, 1455 De Maisonneuve Blvd. W., Montreal, QC H3G 1M8, Canada; thomas.fevens@concordia.ca

**Keywords:** ACL injury, injury prevention, motion analysis, kinematics

## Abstract

Traditional motion analysis systems are impractical for widespread screening of non-contact anterior cruciate ligament (ACL) injury risk. The Kinect V2 has been identified as a portable and reliable alternative but was replaced by the Azure Kinect. We hypothesize that the Azure Kinect will assess drop vertical jump (DVJ) parameters associated with ACL injury risk with similar accuracy to its predecessor, the Kinect V2. Sixty-nine participants performed DVJs while being recorded by both the Azure Kinect and the Kinect V2 simultaneously. Our software analyzed the data to identify initial coronal, peak coronal, and peak sagittal knee angles. Agreement between the two systems was evaluated using the intraclass correlation coefficient (ICC). There was poor agreement between the Azure Kinect and the Kinect V2 for initial and peak coronal angles (ICC values ranging from 0.135 to 0.446), and moderate agreement for peak sagittal angles (ICC = 0.608, 0.655 for left and right knees, respectively). At this point in time, the Azure Kinect system is not a reliable successor to the Kinect V2 system for assessment of initial coronal, peak coronal, and peak sagittal angles during a DVJ, despite demonstrating superior tracking of continuous knee angles. Alternative motion analysis systems should be explored.

## 1. Introduction

Anterior cruciate ligament (ACL) injuries are becoming increasingly common among athletes, with more than a quarter of a million injuries in North America every year [1]. They are an important cause of morbidity and increase the risk of osteoarthritis in the long term, while costing the United States close to USD 7 billion annually [2,3,4]. In response to the increasing burden of ACL injuries, injury prevention programs such as FIFA11+ have been developed and shown to effectively decrease the incidence of ACL injury [5,6]. Furthermore, biomechanics research has demonstrated that specific knee kinematics are associated with increased risk for ACL injury, highlighting the potential for risk screening to identify athletes who can benefit from targeted prevention programs like FIFA11+. In their landmark paper, Hewett et al. (2005) used a high-speed motion analysis system to analyze knee kinematics during the drop vertical jump (DVJ) and found that increased initial and peak coronal abduction angles and decreased peak sagittal angle are associated with increased risk for ACL injury [7].

While the findings of Hewett et al. (2005) provided valuable knowledge in terms of ACL injury risk prognostication, the high cost and impracticality of traditional multi-camera motion tracking systems continued to pose barriers for widespread screening programs. To this effect, studies from Gray and colleagues as well as from our group have identified the Microsoft Kinect V2 (Microsoft, Redmond, WA, USA) as an alternative motion tracking system that does not require markers, and is cost effective, portable, and reliable for assessing the DVJ compared to a gold-standard Vicon motion analysis system (Vicon, Denver, CO, USA) [8,9]. Further, our group has demonstrated that specific DVJ parameters measured by the Kinect V2 have good to excellent prognostic value for ACL injury risk in high-level athletes [10]. However, Microsoft has since discontinued production of the Kinect V2 [11]. The successor to the Kinect V2 is the Azure Kinect system, which is currently available on the market and a potential replacement for the Kinect V2 as a motion tracking system amenable to widespread use in DVJ assessment for ACL injury risk screening. To date, there are no known published findings that evaluate the accuracy of the Azure Kinect in assessing knee angles during the DVJ.

In the present study, we compared the Azure Kinect system to its predecessor, the Kinect V2 system, in terms of reliability for assessing 3-dimensional knee kinematics. Specifically, we investigated the level of agreement in initial coronal angle (IC), peak coronal angle (PC), and peak sagittal angle (PS) between the two systems throughout the DVJ. We hypothesize that the Azure Kinect assesses the aforementioned DVJ parameters with similar accuracy to its predecessor, the Kinect V2.

## 2. Materials and Methods

### 2.1. Participants

The investigation was a descriptive laboratory study. Ethics approval was received by the Research Ethics Board of the McGill University Health Centre (MUHC) prior to the start of this investigation, and informed consent was obtained from all participants in this study. Participants had a moderate-to-high involvement in sports and were recruited from the practice of 2 fellowship-trained orthopedic arthroscopy surgeons. Participants had no lower limb injuries potentially affecting their ability to perform a DVJ. Exclusion criteria included individuals aged less than 18 years old or greater than 50 years old, or individuals who were not cleared by a physician to safely perform a DVJ. Tracking of the participants’ DVJs occurred between 2021 and 2022. Participant characteristics are summarized in Table 1.

### 2.2. Motion Analysis

**Figure 1:**
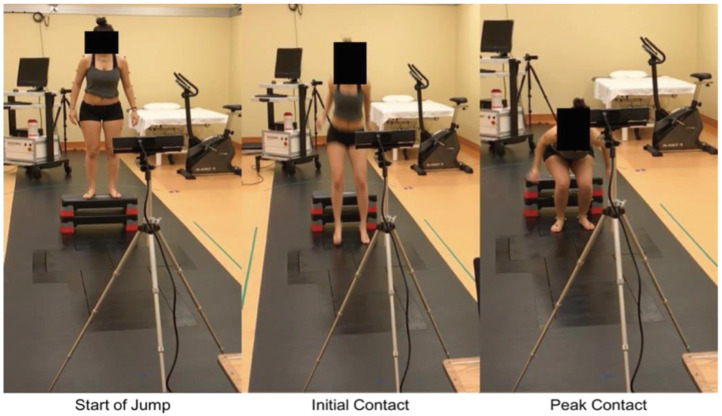
Images of a Kinect V2 setup showing significant time points throughout a DVJ: start of jump, the initial contact, and the peak contact [9].

**Figure 2:**
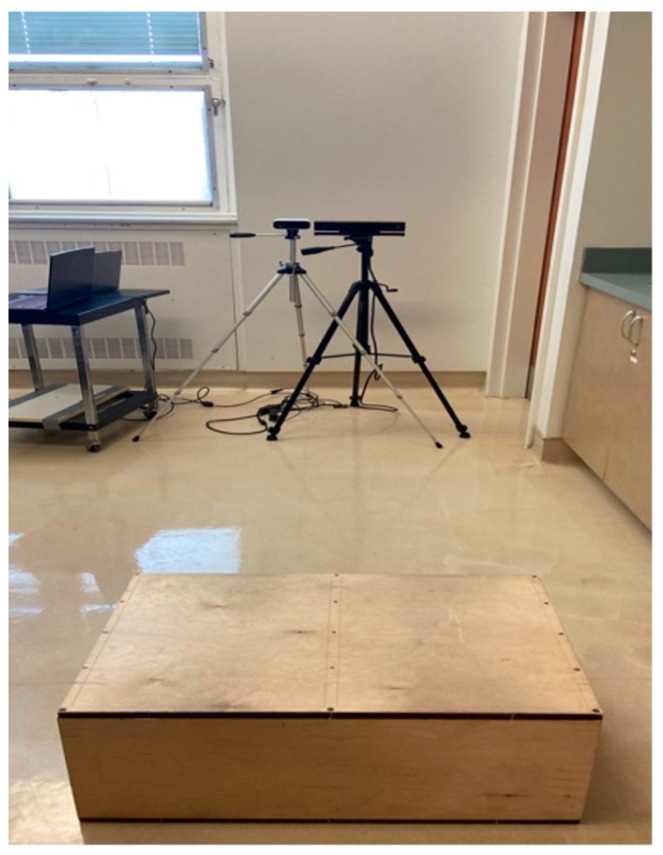
Image displaying the Azure Kinect and Kinect V2 mounted on tripods at a distance of 2.5 m from a 31 cm box for optimal accuracy.

**Figure 3:**
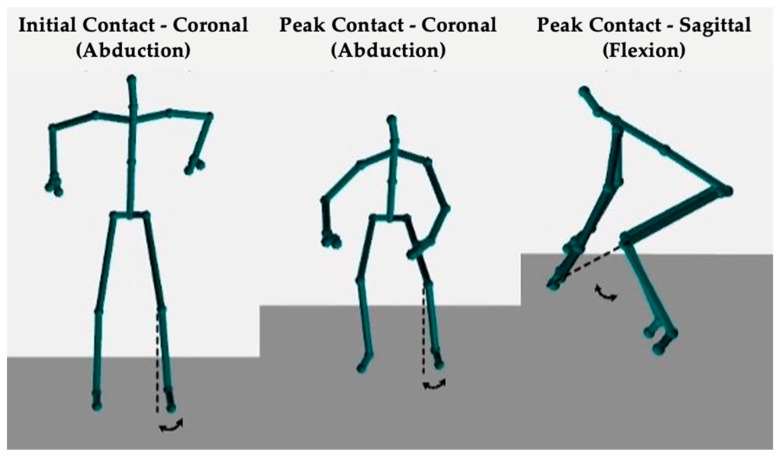
Images of the output from either the Azure Kinect or Kinect V2 depicting the DVJ parameters assessed: IC, PC, and PS angles.

Each participant was instructed on proper technique before completing one familiarization trial and four DVJs, with the majority performing three valid DVJs (Figure 1). Valid DVJs are defined as DVJs performed with appropriate technique and were the only jumps used for analysis. The DVJs were performed with a 31 cm box while assessment of knee kinematics was conducted simultaneously by the Azure Kinect (Microsoft, Redmond, WA, USA, Azure Kinect Sensor SDK 1.4.1, Azure Kinect Body Tracking SDK 1.0.1) and the Kinect V2 (Microsoft, Redmond, WA, USA, Kinect V2 SDK version 2.0), respectively (Figure 2). The DVJ parameters assessed included IC, PC, and PS angles (Figure 3). IC was defined as the coronal knee angle when the participant’s feet first make contact with the floor during the DVJ. PC was defined as the largest coronal knee angle (most valgus knee angle) throughout the entire DVJ after IC. PS was defined as the largest knee flexion angle throughout the entire DVJ.

The Azure Kinect and Kinect V2 both perform 3-dimensional tracking using an infrared (IR) depth sensor with a capture rate of 30 frames per second and utilize the time of flight (ToF) principle for depth estimation [8,12]. Both systems also have an RGB camera, which was not utilized in this study. The IR camera of the Azure Kinect has an improved resolution (1024 × 1024 px) in comparison to the Kinect V2 (512 × 424 px), as well as a wider depth of view [12]. For 3-dimensional motion tracking, the Azure Kinect inputs the data obtained through the infrared depth sensors into a deep learning model trained on large data sets, to extrapolate the position of the subject’s joints; this is also known as a deep neural network (DNN) model. Specifically, they trained a Convolutional Neural Network to extract 2-dimensional joint positions from the infrared image and then use the depth map to infer 3-dimensional values. By contrast, the Kinect V2 utilizes a random forest model which was trained on depth images and their corresponding skeletal joint information, in order to directly output the 3-dimensional positions of the joints [13]. The Azure Kinect body tracking is thus much more computationally expensive and needs extensive datasets for training, due to its reliance on pose estimation methods.

**Figure 4:**
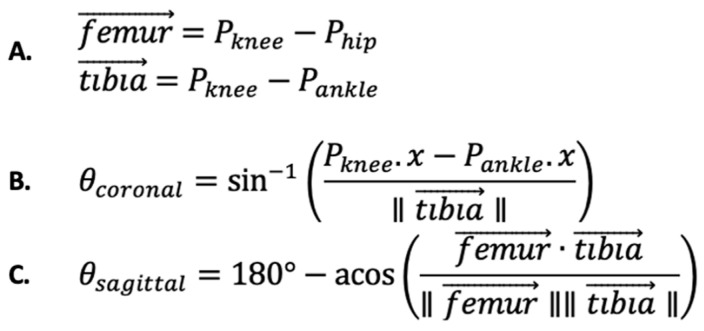
Formulas utilized by our software. (**A**) Vector definition of femur and tibia. (**B**) Formula for calculating coronal knee abduction angles. (**C**) Formula for calculating sagittal knee flexion angles. IC and PC abduction angles are identified based on the angles generated by formula (**B**) throughout the DVJ. PS knee flexion angles are identified based on the angles generated by formula (**C**) throughout the DVJ.

Our software employed a set of formulas that used force vectors from the knee to the hip as a surrogate for the femur and from the knee to the ankle as a surrogate for the tibia (Figure 4A). These formulas provided measurements of knee abduction in the coronal plane (Figure 4B) and knee flexion in the sagittal plane (Figure 4C) at every frame throughout the DVJ. Negative angles in the coronal plane correspond to more knee valgus, while smaller values in the sagittal plane indicate less knee flexion. Subsequently, our software identified the optimal jump frames from which the IC, PC, and PS angles were extracted. To do so, our software was designed to recognize specific movements throughout a DVJ, known as kinematic triggers. The kinematic trigger used to identify the initial contact frame was the moment at which the foot joint stops travelling in the downward direction, indicating that the subject had contacted the floor. The kinematic trigger used to identify the peak abduction frame was the moment the coronal knee angle was at its most valgus. Similarly, the kinematic trigger used to indicate the peak flexion frame was the moment at which the sagittal knee angle was at its highest value. Our software generated an Excel file with IC, PC, and PS angles from both the Azure Kinect and Kinect V2 for further analysis.

### 2.3. Statistical Analysis

The Shapiro–Wilk test for normality was conducted for each parameter. The Paired Samples *t*-test was used to compare normally distributed parameters, while the Wilcoxon Signed Rank test was used to compare non-normally distributed parameters. The level of agreement between the Azure Kinect and Kinect V2 was assessed using the intraclass correlation coefficient (ICC) (Two-Way Mixed, Absolute Agreement, Single Measures). The ICC is a value between 0 and 1, where values below 0.5, between 0.5 and 0.75, between 0.75 and 0.9, and above 0.9 indicate poor, moderate, good, and excellent reliability, respectively [14]. *p* < 0.05 was interpreted as statistically significant. The Paired Samples *t*-test, Wilcoxon Signed Rank test, and ICC analyses were performed using SPSS Version 27 (IBM, Armonk, NY, USA).

A post hoc power analysis was conducted using G*Power version 3.1.9.7 [15] to determine the minimum sample size required for the present study. Results indicated the required sample sizes to achieve 80% power for detecting a medium effect, at a significance criterion of α = 0.05, were *N* = 34 for the Paired Samples *t*-test and *N* = 35 for the Wilcoxon Signed Rank test. The required sample size to achieve 80% power for detecting an ICC value greater than 0.3 was *N* = 66 [16]. Thus, the obtained sample size of *N* = 69 was adequate for the present study.

## 3. Results

A total of 69 participants were recruited for this investigation. Each participant performed four DVJs, with the majority performing three valid DVJ (58 participants, 84%), amounting to a total of 206 jumps and 412 data points for each knee angle. The analysis was performed for each DVJ parameter for each leg separately.

Based on the Shapiro–Wilk test, only PC angle for the left knee was normally distributed for both systems (W = 0.987 and 0.991, *p* = 0.06 and 0.26, respectively, for Azure Kinect and Kinect V2). As such, the Paired Samples *t*-test was performed to compare the mean left PC angle measured by the two systems. The Wilcoxon Sign Rank test was used to compare the mean IC and PS angles for both knees and mean PC angle for the right knee between the Azure Kinect and Kinect V2. The mean angles measured by the Azure Kinect and Kinect V2, along with their differences, are presented in Table 2. The Azure Kinect consistently measured smaller IC angles compared to the Kinect V2 with a statistically significant difference of 0.51 and 1.12 degrees for the left and right knee, respectively. The Azure Kinect also reported smaller PC angles; however, this difference was not statistically significant. Mean PS angles assessed by the Azure Kinect were significantly smaller than mean PS angles assessed by the Kinect V2, with differences of −13.84 and −12.33 degrees for the left and right knee, respectively. The Azure Kinect tracked each parameter with smaller standard deviations compared to the Kinect V2.

Subsequently, intraclass correlation coefficient analysis was performed to further evaluate the level of agreement on DVJ parameters between the Azure Kinect and the Kinect V2. The ICC values are presented in Table 3. Based on standard ICC interpretation [14], there was poor agreement between the two systems for left and right knee IC angles, as well as for left and right knee PC angles. There was moderate agreement for left and right knee PS angles. All ICC values reported were statistically significant.

**Figure 5:**
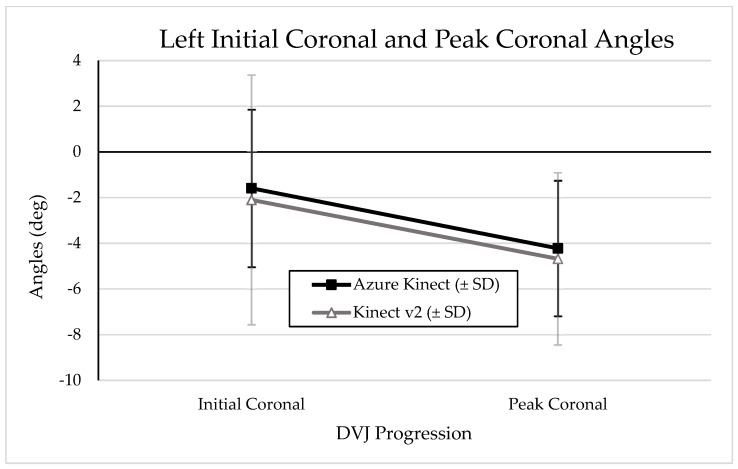
Graphical representation of mean left knee IC and PC angles with standard deviations, as measured by the Azure Kinect and Kinect V2. More negative values indicate more knee valgus.

**Figure 6:**
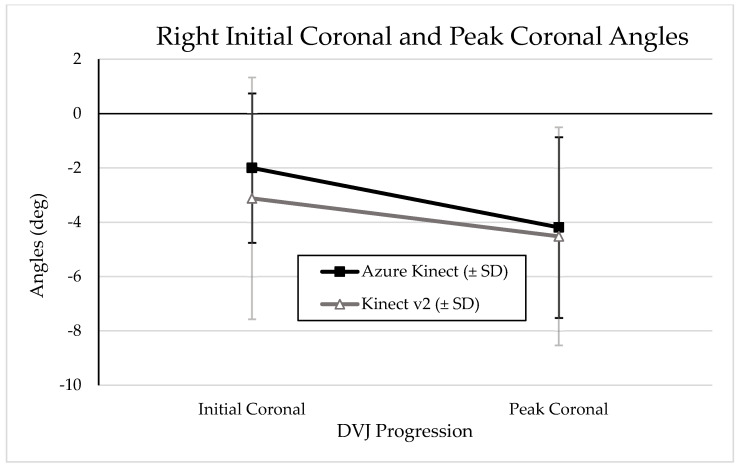
Graphical representation of mean right knee IC and PC angles with standard deviations, as measured by the Azure Kinect and Kinect V2. More negative values indicate more knee valgus.

**Figure 7:**
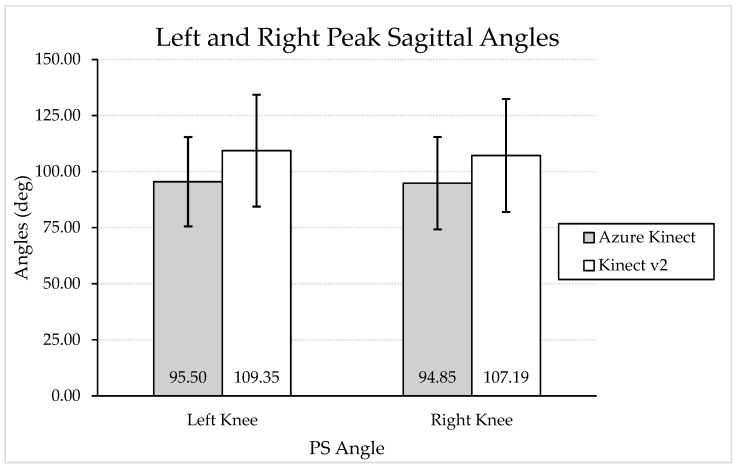
Graphical representation of mean left and right knee PS angles with standard deviation bars, as measured by the Azure Kinect and Kinect V2. Smaller values indicate less knee flexion.

Graphical representations of the change from IC to PC angles during a DVJ as measured by the Azure Kinect and Kinect V2 are depicted in Figure 5 and Figure 6 for left and right knees, respectively. A graphical representation of mean PS angles measured by the Azure Kinect and Kinect V2 is depicted in Figure 7. Visual analysis of the graphical representations shows that the Azure Kinect consistently measured smaller absolute values, with smaller standard deviations, than the Kinect V2 across all knee angles.

**Figure 8:**
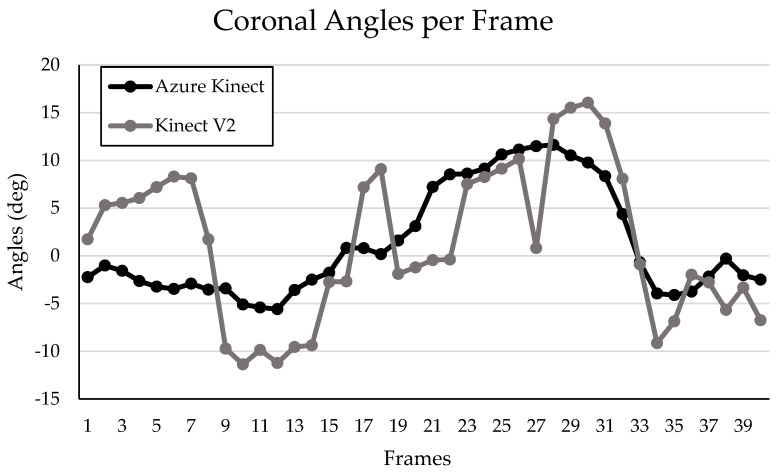
Left knee coronal angles per frame of a sample participant during a DVJ measured by the Kinect V2 and Azure Kinect.

**Figure 9:**
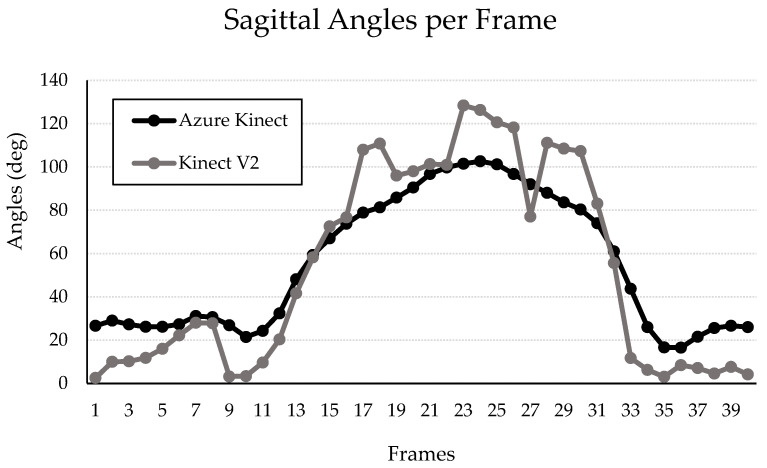
Left knee sagittal angles per frame of a sample participant during a DVJ measured by the Kinect V2 and Azure Kinect.

Graphical representation of the frame-by-frame coronal and sagittal knee angles measured throughout a single DVJ by both the Kinect V2 and the Azure Kinect systems are presented in Figure 8 and Figure 9, respectively. The two graphs demonstrate the overall measurement pattern of the Kinect V2 and the Azure Kinect systems. The measurement pattern for the Kinect V2 system displays a higher degree of variation in both the coronal and sagittal planes throughout the DVJ, compared to the Azure Kinect (Figure 8 and Figure 9).

## 4. Discussion

This investigation was the first to compare the Azure Kinect to its predecessor motion analysis system, the Kinect V2, for measuring IC, PC, and PS angles during a DVJ. Considering that the Kinect V2 has previously been tested and validated for assessing the DVJ in comparison to a gold-standard Vicon system, this study directly compared the Azure Kinect with the Kinect V2 [8,9]. Contrary to our hypothesis, we observed that the Azure Kinect consistently reports smaller IC and PS angles while demonstrating poor-to-moderate agreement for IC, PC, and PS angles with its predecessor, the Kinect V2.

As previously discussed, a growing body of literature on knee kinematics as risk factors for ACL injury has generated a need for cost-effective and practical motion analysis systems that are amenable to use in large scale risk screening [7,17,18]. Hewett et al.’s paper observed that increased IC and PC abduction and decreased PS angles were associated with increased risk for non-contact ACL injury in female varsity athletes. It is thus particularly concerning that the Azure Kinect reported significantly less valgus knee angles at initial contact, with a difference of 0.51 and 1.12 degrees for the left and right knee, respectively, in comparison to the Kinect V2. Taken together, the findings in the present investigation and those of Hewett et al.’s study suggest that the use of the IC angles obtained from the Azure Kinect would likely lead to the classification of more athletes as low risk for ACL injury, decreasing the sensitivity of an Azure Kinect-based ACL injury risk assessment tool. Similarly, when taken together, the findings in the present investigation and those of Hewett et al.’s study suggest that the use of the PS angles obtained from the Azure Kinect would likely lead to the classification of fewer athletes as low risk for ACL injury, decreasing the specificity of an Azure Kinect-based ACL injury risk assessment tool. Of note, the Azure Kinect also reported smaller PC angles on average; however, this difference was not statistically significant. Thus, ACL injury risk assessment based on PC angles obtained from the Azure Kinect may also classify more high-risk athletes as low risk, decreasing sensitivity.

Overall, ICC analysis showed poor agreement between the Azure Kinect and the Kinect V2 for IC and PC angles (ICC values ranging from 0.135 to 0.446). There was minimally improved agreement in assessing knee angles in the sagittal plane, with ICC values of 0.688 and 0.605 for the left knee and right knee, respectively. Ultimately, these results suggest that the Azure Kinect system does not report IC, PC, and PS measurements during a DVJ in a similar pattern to the Kinect V2, the latter of which has previously been validated for measuring knee angles during a DVJ [8,9]. There is therefore a need to either improve the Azure Kinect system’s accuracy for measuring IC, PC, and PS knee angles during a DVJ or to identify other cost-effective and practical motion analysis systems that are more suitable to the task.

While this study observed suboptimal reliability when using the Azure Kinect to assess the aforementioned knee angles during a DVJ, other studies have found good reliability in other domains of motion tracking. In a pilot study on five young and healthy subjects, Albert et al. (2020) found that the Azure Kinect had significantly higher accuracy in assessing spatial gait parameters compared to the Kinect V2, citing improved hardware and motion-tracking algorithms as major factors [12]. Similarly, Antico et al. (2021) conducted a study comparing the Azure Kinect to the Vicon system for postural assessment of 26 healthy subjects and found that the Azure Kinect provided very accurate tracking of the main body joints [19]. Of note, the ICC value between the Azure Kinect and the Vicon for tracking the sternum, hand, and trunk during lateral reach and forward reach exercises were all above 0.9, indicating excellent agreement. These ICC values were higher than the ICC values obtained when comparing the Kinect V2 and the Vicon for the same movements and anatomical landmarks in a similar study by Clark et al. (2012) [20]. While these findings suggest that the Azure Kinect is superior to the Kinect V2 with regards to three-dimensional tracking, it is worth noting the different exercises tracked in each study. The study by Albert et al. asked participants to walk at relatively slow speeds (between 3 and 4.7 kmh^−1^), while Antico et al. focused on simple upper body movements such as lateral reach and forward reach with one arm [12,19]. Also, Antico et al. identified the main limitations of the Azure Kinect to be in tracking quick movements and movements along the focal axis. In the present study, assessment of PS angles during the DVJ involves tracking the knees along the focal axis as they move towards the sensors at relatively high speed when the knees bend. In considering the findings of the present study within this context, it is possible that the Azure Kinect is reliable for assessment of simple movements at a moderate pace, whereas it has limited accuracy when tracking quick movements of the lower limbs such as in the DVJ.

It is particularly surprising that the Azure Kinect demonstrates such little agreement with the Kinect V2 in assessing DVJ parameters, considering that they are both built with similar hardware consisting of an RGB camera and infrared depth sensor; moreover, these sensors have better resolution in the Azure Kinect compared to the Kinect V2 [12]. The wide range of accuracy that the Azure Kinect demonstrates in assessing movements of different complexities and involving different areas of the body may be explained in part by its dependence on DNN machine learning for interpreting three-dimensional kinematics. With machine learning, the system in question generally improves its accuracy as it interprets more data. As such, the Azure Kinect should be more accurate than its predecessor when interpreting simple and commonly encountered movements such as walking on a treadmill or performing lateral and forward reach movements, where it has likely been exposed to enough data to sufficiently interpret such movements [12,19]. Conversely, the Azure Kinect may be disadvantaged in interpreting more complex movements of the body that are not commonly seen in a variety of exercises, particularly if the machine learning model has not encountered enough of such data. We hypothesize that the Azure Kinect DNN has not been exposed to sufficient data in which movements resemble the DVJ, due to limited distribution of the Azure Kinect as a result of global chip shortages and other challenges relating to the COVID-19 pandemic. As such, it is possible that the Azure Kinect mistakenly attempts to correct “abnormal” kinematic outputs, such as increased knee valgus angles. A similar situation may partially explain the discrepancies seen with sagittal knee angles. By extension, there would intuitively be slightly less discrepancy between the Azure Kinect and the Kinect V2 as sagittal knee flexion is a movement more commonly seen in other exercises such as walking on the treadmill. This hypothesis is reflected in the summary graphical representations of IC, PC, and PS angles in this investigation (Figure 5 and Figure 6).

Possible over-correcting of coronal and sagittal knee angles due to insufficient exposure to DVJ-like data would also explain the decreased standard deviations, and thus narrower spread of angles, that was observed for all parameters when measured by the Azure Kinect in comparison to the Kinect V2. However, it is possible that the smaller standard deviations indicate that the Azure Kinect measures IC, PC, and PS knee angles during a DVJ with greater precision, although this is highly dependent on the actual extent of spread of each parameter, which was not obtained from a gold-standard motion analysis system in this study. Furthermore, smaller standard deviations do demonstrate that the Azure Kinect is, overall, more consistent than the Kinect V2. The increased variability of the Kinect V2 compared to the Azure Kinect when measuring knee angles throughout a DVJ is also highlighted in the measurement patterns displayed in Figure 8 and Figure 9. Therefore, our data suggest that the Azure Kinect may be better suited than the Kinect V2 for measuring continuous data. These results are in line with other investigations discussed above, where the Azure Kinect demonstrated higher accuracy compared to the Kinect V2 when measuring spatial gait parameters and tracking main body joints [12,19]. Considering these findings, perhaps the Azure Kinect would be more appropriate than the Kinect V2 for ACL injury risk prognostication using continuous variables measured during a DVJ.

Moreover, it could be beneficial to re-visit the accuracy of the Azure Kinect for measuring IC, PC, and PS knee angles during the DVJ in the future, if the DNN were to be exposed to more kinematic data related to the DVJ. However, whether this exposure and machine learning takes place is largely dependent on many factors, including chip shortages and the future directions of the Microsoft company. Other motion analysis systems that are available on the market and of similar cost and practicality may be explored, such as the Intel RealSense Depth Camera (Intel, Santa Clara, CA, USA) or Structure Core sensors (Occipital, San Francisco, CA, USA). One challenge is the absence of a proprietary skeletal tracking system, which exists for the Azure Kinect.

While the current investigation presents important findings for the future direction of DVJ screening for ACL risk assessment, there exist some potential limitations that merit discussion. Firstly, a sex-specific analysis would have been optimal to account for anatomical differences and any possible associated variations in jumping patterns. Due to the low number of female athletes in our sample, conducting a sex-specific analysis was not possible without a significant decrease in power. Considering that the DVJs were all performed with the same technique, small potential differences in jumping patterns would likely have minimal influences on the results of this study. Secondly, considering that it was not possible to have the Azure Kinect and Kinect V2 cameras in the exact same position while simultaneously tracking each DVJ, the slight difference in the point of view between the two systems may have increased the discrepancy in knee angle assessment throughout each DVJ. However, we believe that this had a relatively minor effect, as the two systems were placed side by side with less than 1 cm between their respective sensors, while the participant was 2.5 metres away from both sensors. It is worth noting, however, that Yeung et al. (2021) found that the Azure Kinect had better tracking performance than the Kinect V2 on participants walking on a treadmill when placed at non-frontal viewing angles (22.5°/45°/67.5°/90°), while the Kinect V2 performed better at frontal viewing angle (0°) [21]. As such, future studies should assess the accuracy of the Azure Kinect in comparison to the Kinect V2 for assessing DVJs from various viewing angles.

Another potential limitation of this study is the degree of error inherent in the Kinect V2, and the theoretical risk that the discrepancy in mean DVJ parameters is a result of the Azure Kinect being more accurate than the Kinect V2. However, this is unlikely, considering that the Kinect V2 has previously been validated for measuring knee angles during a DVJ in comparison to the gold-standard Vicon system [8,9].

In considering the findings of the present investigation, further research on DVJ parameters would likely benefit from utilizing other motion tracking systems alongside the Azure Kinect and tracking the DVJ from various viewing angles. Moreover, further research on the accuracy of the Azure Kinect compared to the Vicon when measuring DVJ parameters should be conducted following Microsoft’s next update. These conditions can be beneficial for identifying the optimal motion tracking conditions and alternative motion analysis systems for the DVJ, and ultimately for ACL injury risk assessment.

## 5. Conclusions

At the present time, the Azure Kinect is not a reliable replacement for the Kinect V2 when assessing IC, PC, and PS angles during a DVJ, despite demonstrating superior tracking of continuous angles throughout the DVJ. Further exposure of the Azure Kinect’s DNN to DVJ data may improve the accuracy of this system. There exists a need to identify optimally cost-effective and accurate motion capture systems for DVJ assessment and, by extension, for DVJ-based ACL injury risk assessment.

## Figures and Tables

**Table 1:** Participant characteristics: sex, age, height, weight, and BMI. Data are shown as mean and standard deviation.

	Male Participants	Female Participants	All Participants
N	49	20	69
Age	26.6 ± 7.05	27.96 ± 6.63	27.59 ± 6.73
Height (m)	1.68 ± 0.08	1.78 ± 0.06	1.76 ± 0.08
Weight (kg)	67.72 ± 13.31	81.73 ± 11.55	77.67 ± 13.59
BMI (kg/m^2^)	23.80 ± 3.93	25.60 ± 3.30	25.07 ± 3.56

**Table 2:** Results of the Wilcoxon Signed Rank Test and Paired Samples *t*-Test conducted on the IC, PC, and PS angles measured by the Azure Kinect system and the Kinect V2 system. Negative values in the coronal plane represent increased knee valgus while smaller values in the sagittal plane represent decreased knee flexion.

	**Mean Angle ± SD (°)**			**Wilcoxon Signed** **Rank Test**
**Knee Angle**	**Azure Kinect**	**Kinect V2**	**Difference**	***p*** **Value**
Left knee IC	−1.59 ± 3.45	−2.1 ± 5.46	0.51	<0.01
Right knee IC	−2.00 ± 2.76	−3.12 ± 4.45	1.12	<0.01
Right knee PC	−4.19 ± 3.33	−4.52 ± 4.02	0.32	0.16
Left knee PS	95.5 ± 19.90	109.35 ± 24.93	−13.84	<0.01
Right knee PS	94.85 ± 20.61	107.19 ± 25.23	−12.33	<0.01
	**Mean Angle ± SD (°)**			**Paired Samples** ***t*-Test**
**Knee Angle**	**Azure Kinect**	**Kinect V2**	**Difference**	***p*** **Value**
Left knee PC	−4.22 ± 2.97	−4.68 ± 3.77	0.46	0.07

**Table 3:** ICC results for knee IC, PC, and PS angles measured by the Azure Kinect system and the Kinect V2 system. ICC calculations were performed using the following condition: Two-Way Mixed, Absolute Agreement, Single Measures.

		95% Confidence Interval		F Test with True Value 0			
Knee Angle	Intraclass Correlation	Lower Bound	Upper Bound	Value	*df1*	*df2*	*p* Value
Left knee IC	0.135	−0.001	0.266	1.313	205	205	0.03
Right knee IC	0.186	0.055	0.311	1.48	205	205	<0.01
Left Knee PC	0.442	0.326	0.545	2.60	205	205	<0.01
Right knee PC	0.446	0.33	0.549	2.61	205	205	<0.01
Left knee PS	0.608	0.185	0.792	6.20	205	205	<0.01
Right knee PS	0.655	0.284	0.813	6.94	205	205	<0.01

## Data Availability

Data are available upon request from the authors.
